# No Overt Deficits in Aged Tau-Deficient C57Bl/6.*Mapt^tm1(EGFP)Kit^* GFP Knockin Mice

**DOI:** 10.1371/journal.pone.0163236

**Published:** 2016-10-13

**Authors:** Annika van Hummel, Mian Bi, Stefania Ippati, Julia van der Hoven, Alexander Volkerling, Wei S. Lee, Daniel C. S. Tan, Andre Bongers, Arne Ittner, Yazi D. Ke, Lars M. Ittner

**Affiliations:** 1 Dementia Research Unit, Department of Anatomy, School of Medical Sciences, Faculty of Medicine, University of New South Wales, Sydney, NSW, Australia; 2 Biological Resources Imaging Laboratory, Mark Wainright Analytical Centre, University of New South Wales, Sydney, NSW, Australia; 3 Motor Neuron Disease Unit, Department of Anatomy, School of Medical Sciences, Faculty of Medicine, University of New South Wales, Sydney, NSW, Australia; 4 Neuroscience Research Australia, Sydney, NSW, Australia; Nathan S Kline Institute, UNITED STATES

## Abstract

Several mouse lines with knockout of the tau-encoding *MAPT* gene have been reported in the past; they received recent attention due to reports that tau reduction prevented Aβ-induced deficits in mouse models of Alzheimer’s disease. However, the effects of long-term depletion of tau *in vivo* remained controversial. Here, we used the tau-deficient GFP knockin line *Mapt*^*tm1(EGFP)kit*^ on a pure C57Bl/6 background and subjected a large cohort of males and females to a range of motor, memory and behavior tests and imaging analysis, at the advanced age of over 16 months. Neither heterozygous nor homozygous *Mapt*^*tm1(EGFP)kit*^ mice presented with deficits or abnormalities compared to wild-type littermates. Differences to reports using other tau knockout models may be due to different genetic backgrounds, respective gene targeting strategies or other confounding factors, such as nutrition. To this end, we report no functional or morphological deficits upon tau reduction or depletion in aged mice.

## Introduction

Alzheimer’s disease (AD) is the most common neurodegenerative disease, characterized by a progressive decline of cognition due to loss of synapses and neurons. For comparison, Frontotemporal dementia (FTD) is the second most prevalent form of dementia occurring before the age of 65, comprising a heterogeneous group of neurodegenerative conditions. In AD, and in approximately half of FTD brains, the microtubule-associated protein tau becomes hyperphosphorylated and forms intracellular fibrillar inclusions. These tau-containing neurofibrillary tangles are found together with extracellular amyloid-β (Aβ) plaques, forming the neuropathological hallmarks of the disease [1].

The human tau protein is encoded by the *MAPT* gene on chromosome 17 and comprises several functional regions in the primary polypeptide sequence [2]; the N-terminal half includes a proline-rich region that mediates interaction with partner proteins, including Src-kinase, followed by multiple microtubule-binding (MTB) repeats and a C-terminal tail region of unknown function. Alternative splicing of exons 2, 3 and 10 results in different isoforms expressed in the adult human brain, containing either 0, 1 or 2 N-terminal inserts (0-2N) and 3 or 4 MTB repeats (3/4R). Tau is enriched in the axon of neurons, but also found in other compartments including dendrites and post-synapses [3]. Tau contributes to microtubule dynamics, regulation of motor-driven transport along microtubules and synaptic signaling [4].

Given its role in neurodegeneration and several physiological processes in neurons, one may have expected that depletion of tau results in significant functional and/or developmental deficits in *tau*^*–/–*^mice. However, two independent studies reported no overt phenotypes in their respective *tau*^*–/–*^lines in young mice [5, 6]. Two further *tau*^*–/–*^lines were generated, only one of which has undergone functional testing [7, 8]. The lack of an overt phenotype in *tau*^*–/–*^mice is likely due to compensation by other MAPs [9]. Interestingly, hyperactivity and muscle weakness with reduced motor coordination were reported subsequently for *tau*^*–/–*^mice [10], and reduced motor coordination and locomotion after 1 year of age [11]. This was contrasted by only subtle motor deficits in *tau*^*–/–*^mice in a subsequent study [12]. Furthermore, normal spatial memory was found in young to middle-aged *tau*^*–/–*^mice [10, 13], with deficits developing only in aged mice [14]. Finally, crossing *tau*^*–/–*^mice with amyloid-β-forming lines modelling AD prevented memory deficits and improved survival in independent reports [3, 13], but led to enhanced axonal pathology in another study [15]. Hence, findings from different *tau*^*–/–*^lines remain conflicting.

Given the importance of *tau*^*–/–*^mice for AD research and their frequent use in crossings with other lines, a detailed understanding of possible deficits in aged mice is required. Here, we report absence of overt functional and morphological deficits in hetero- and homozygous mice of advanced ages (>16 months), from one of the *tau*^*–/–*^lines previously generated, the *Mapt*^*tm1(EGFP)kit*^ GFP (*tau*^*GFP/GFP*^) knockin strain [7].

## Materials and Methods

### Mice

*Mapt*^*tm1(EGPF)kit*^ (here referred to as *tau*^*GFP/GFP*^) mice have been generated previously (Jax Mice #004779) [7]. *Tau*^*GFP/GFP*^ were obtained and then maintained for >20 generations on a C57Bl/6J background. Mice were group housed in individually ventilated cages on a 12h light/dark cycle. Mice were housed in groups of 3 to 6 littermates in individually ventilated cages (Allentown, USA) in a special pathogen free facility. Cages contained Anderson's 1/8th inch corn cob bedding (Tecniplast, Italy) and were enriched with wooden chew sticks, red transparent plastic houses and paper nesting material. They had access to water and chow containing 3.5g/kg omega-3 fatty acid derived from fishmeal *ad libitum* (Mouse Breeder Diet, Grodon’s Specialty Stockfeeds, Australia). Heterozygous *tau*^*GFP/GFP*^ males and females were mated to obtain all desired genotypes and littermate controls. Both males and females were used in this study. Mice were aged to 16 months of age before being subjected to behavioral testing, and were euthanized at 19–22 months of age for further analysis of tissue. All animal experiments were approved by the Animal Ethics & Care Committee of the University of New South Wales.

### Behavioral, memory and motor testing

The following behavioral tests all used the same cohort of 16 month old mice: homozygous *tau*^*GFP/GFP*^ males (n = 8) and females (n = 12); heterozygous *tau*^*GFP/+*^ males (n = 18) and females (n = 20); *tau*^*+/+*^ littermate control males (n = 12) and females (n = 15), unless otherwise stated.

*Rota-Rod*: Motor performance of mice was determined using a Rota-Rod (Ugo Basile) in acceleration mode (5-60rpm) over 120 seconds, as previously reported [15]. The longest time each mouse remained on the turning wheel out of 5 attempts per session was recorded.

*Hanging Wire Test*: Mice were placed on a rectangular wire mesh (Aldi) and slowly flipped over such that they hung upside down over a Perspex box. Mice were allowed to hang upside down for a maximum of three minutes, and latency to falling off was recorded (longest time out of 2 attempts).

*Morris Water Maze*: Spatial memory testing was done as previously reported [16]. Briefly, the apparatus consisted of a 1.2m diameter tank with a 40cm high Perspex platform (diameter 10cm), which was placed roughly 20cm from the edge of the wall. The tank was filled to 0.5-1cm above the surface of the platform and a non-toxic, acrylic-based paint was added to the water to obscure the platform. Four signposts with different shapes were placed equidistant around the pool as visual cues. Mice were acclimatized to the room for 1 hour prior to testing each day. Days 1 to 7 consisted of an acquisition phase, in which mice were placed in the quadrant opposite the platform at one of four starting positions and given 60 seconds to locate the hidden platform. Mice that failed to find the hidden platform were guided to the escape platform and all mice remained on the platform for an additional 60 seconds before being removed from the maze. Mice had four trials per day, each starting from a different position, and the order of starting positions was altered each day. On the eighth day, the platform was removed and the mice were given 30 seconds to explore the pool (probe trial). On the ninth day, the platform was placed back in the pool with a flag attached, and visual cues were removed from outside of the pool, to ensure that all mice had normal vision. Videos were analyzed using the AnyMaze tracking software.

*Elevated Plus Maze*: Anxiety/disinhibition behavior was tested as previously described [16]. Briefly, the Elevated Plus maze (Ugo Basile) consisted of two open and two closed arms (each 35cm x 5.5cm), as well as a central platform (5.5cm x 5.5cm), elevated 60cm above the ground. Mice were acclimatized to the room for 1 hour prior to testing, then placed on the center platform facing an open arm and recorded for 5 minutes. Videos were analyzed using the AnyMaze software.

*Open Field*: Locomotor behavior was tested as previously described [16]. Briefly, mice were placed at the periphery of a40 cm x 40cm Perspex box in an enclosed cupboard and videoed for 10 minutes. Videos were analyzed using the AnyMaze software, and the box divided into an outer and inner zone (inner zone was a 17.5 cm x 17.5 cm square in the center of the box).

*Pole test*: Motor coordination and strength was tested as previously described [17]. Briefly, mice were placed on the top of a 40cm vertical beam (1cm diameter), facing upwards. Time to turn and descend to the bottom was recorded.

### Magnetic Resonance Imaging (MRI)

MRI was done as previously described [16]. Briefly, 3 mice per genotype and gender were anesthetized and transcardially perfused with phosphate buffered saline followed by cold 4% paraformaldehyde (PFA). Brains and tissues were removed and post-fixed in 4% PFA overnight. Prior to imaging the fixed brains were immersed in 9g/L NaCl/H_2_O solution for 24-48h at 21°C to resolve fixation residues in the brain tissue. The brains were then transferred into a 1.3mm ID, 2ml Cryovial (Greiner) and submersed in Perfluoro-Polyether Fomblin^™^ 6Y (Sigma) for susceptibility matching. The Cryovial was then mounted on the tip of a plastic tube which was attached to the automatic positioning system of the MRI system.

To obtain quantitative information about local iron content in the mouse brain samples we applied T2* relaxometry methods as MRI biomarker. R2* has been shown to correlate strongly with local iron deposits and R2* mapping has been established and validated by multiple previous studies [18, 19]. Imaging was performed in a 9.4-T Bruker BioSpec 94/20 Avance III micro-imaging system (Bruker, Ettlingen, Germany) which was equipped with BGA-12S HP gradients with maximum strength 660 mT/m and slew rate 4570 Tm/s and a dedicated 15mm Quadrature Receive/Transmit RF-coil (Bruker, Ettlingen, Germany).

Prior to imaging for each sample field homogeneity was optimized to 2^nd^ order spherical harmonic shims by mapping local field variations using a gradient double echo sequence. A T2* relaxometry dataset and anatomical images were acquired using an isotropic 3D Multi gradient echo sequence (MGE) pulse sequence that has been optimized for small sample imaging at the specific relaxation times. 106 partitions were acquired in coronal slab orientation with 28 gradient echos covering an echo time range of 2.7 to 89ms with the following major parameters: First TE = 2.7ms, ΔTE = 3.45ms, #echos = 28, TR = 100ms, FA = 30°, FOV = 15 × 15 × 8 mm, matrix = 200 × 200× 106, Image Resolution = 75μm^3^ (isotropic), Eff. Spectral BW = 78125Hz, Total acquisition time with 2 ADC averages: 1h and 46 min per specimen. The decay data series was converted from a Bruker proprietary format into 28 individual 3D image blocks in 32-bit Nifti format. To correct for small image shifts from eddy currents individual echo images were re-aligned prior to data fitting. T2* decay curves per region of interest were generated in R (R Core Team, version 3.2.3) and RStudio (Rstudio Team, version 0.99.491) with nonlinear least squares fitting to a mono-exponential decay model, using the base package function *nls*(), to the equation $\frac{N_{t}}{N_{0}}\;=\;ae^{-\lambda t}$ with starting values of *a* = 1 and *λ* = 0. R2* relaxation rate (*λ*) per voxel was calculated using a mono-exponential decay model with a linear fit, using base package function *lm*(), to the equation $log\left(\frac{N_{t}}{N_{0}}\right)\;=\;-\lambda t$. These values were used to generate the R2* relaxation rate maps. *t* represents echo time as described above, *N*_0_ and *N*_*t*_ represent the echo time-series vectors in each image voxel at the first echo (*t* = 2.7*ms*) and the echo vectors at time point *t* respectively. Total brain volume was obtained from the first time point for each sample using 3D Slicer software (NIH).

### Histological analysis

Mice were anesthetized and transcardially perfused with phosphate buffered saline followed by cold 4% paraformaldehyde. Brains were removed and post-fixed in 4% paraformaldehyde for immunohistochemical analysis. Brains were embedded in an 8% agarose gel and sectioned at 50μm on a vibratome (Campden, UK) and free-floating sections were then permeabilized for 20 minutes by incubation in 0.5% NP-40 (Sigma) in standard phosphate buffered saline (PBS) buffer, pH7.4. Following blocking in blocking buffer (3% (v/v) goat serum (Sigma), 2% (w/v) bovine serum albumin (Sigma), 0.1% (v/v) Triton X-100 (Sigma) in PBS), slices were incubated in a primary TH antibody (in blocking buffer 1:1000, Millipore) at 4°C overnight. After washing, slices were incubated in secondary biotinylated antibodies (1:1000 in blocking buffer) for 4 hours, and then washed again. Then, sections were incubated with avidin- HRP-coupled complexes (1:00 vectastain Elite ABC HRP Kit) for 1 hour. Following washing, sections were incubated in a DAB/H_2_O_2_ solution (dilution of one DAB tablet in 15ml PBS as per the manufacturer’s instructions; Sigma) for approximately 10 minutes. Finally, sections were washed, transferred on glass microscopy slides, dried overnight and mounted in DPX (Sigma). Washing of sections was done in PBS containing 0.1% (v/v) Triton X-100. All incubations were done at room temperature on a horizontal shaker at 70rpm in 1ml buffer volume unless indicated otherwise. An Olympus BX51 microscope equipped with a graticulated ocular (U100H6; Olympus) and a DP70 CCD camera was used for bright field imaging and stereological counting. The numbers of neurons within the substantia nigra pars compacta (SNpc) were estimated using an optical fractionator design as previously described [20]. Briefly, TH-positive neurons in the SNpc were distinguished by anatomical location and counted on 50μm thick sections using the graticulated ocular with an optical frame of 50μm by 50μm (125,000μm^3^) on a 20x objective. Between 20–30 such regions were analyzed. Estimated numbers of TH-positive neurons for each mouse was calculated using a SNpc volume of 0.392mm^3^, derived from volumetric calculations of serial sections based on the Allen Mouse Brain Atlas (http://mouse.brain-map.org/).

### Western blotting

Western blotting was done as previously reported [21]. Primary antibodies were against tau (Tau-5, 1:1000, #AHB0042, Invitrogen), Gapdh (1:1000, #MAB374, EMD Millipore), GFP (1:500, #sc-9996, SantaCruz), NR1 (1:1000, #MAB363, EMD Millipore), NR2B (1:1000, #AB1557, EMD Millipore), PSD-95 (1:1000, #MAB1596, EMD Millipore) and drebrin (1:1000, #D3816, EMD Millipore).

### Statistical analysis

All statistical analysis was done using the Graphpad Prism 6.0 software and Student’s t-tests or ANOVA (group analysis). P values of below 0.05 were considered significant. All values are presented as mean and standard error of the mean.

## Results

### *Tau*^*GFP/GFP*^ mice show no gross morphological phenotype

**Fig 1:**
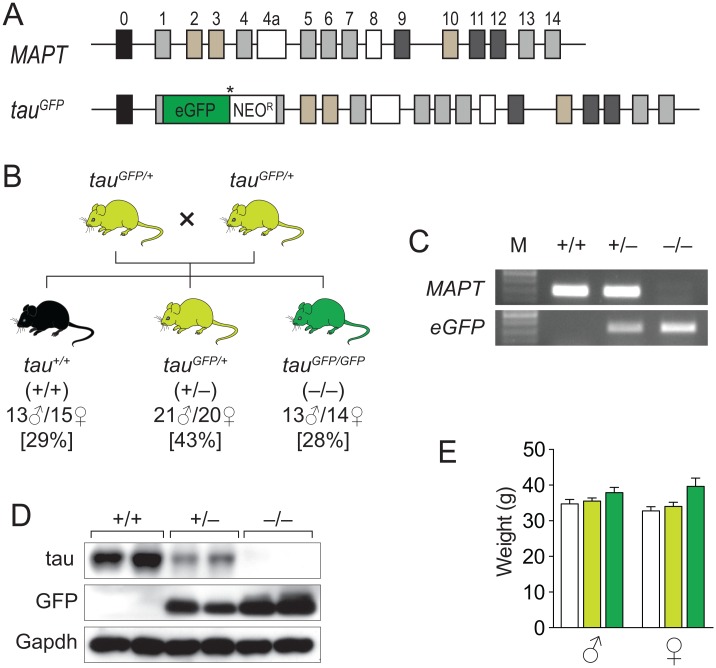
Normal body weight in aged *tau*^*GFP/GFP*^ mice. (A) Knockin strategy of *eGFP* into the *MAPT* locus of *tau*^*GFP/GFP*^ mice (adopted from Tucker et al. 2001). (B) Breeding strategy to obtain aged *tau*^*GFP/GFP*^, *tau*^*+/GFP*^ and *tau*^*+/+*^ mice. All genotypes were obtained at Mendelian distribution as indicated as % of total mice. (C) Genotyping of *MAPT* and *eGFP* in *tau*^*GFP/GFP*^ (–/–), *tau*^*+/GFP*^ (+/–) and *tau*^*+/+*^ (+/+) mice. (D) Western blotting of *tau*^*GFP/GFP*^, *tau*^*+/GFP*^ and *tau*^*+/+*^ brain extracts, showing reduction/loss of tau and expression of GFP in *tau*^*+/GFP*^ and *tau*^*GFP/GFP*^ mice. (E) No significant differences in body weight between *tau*^*GFP/GFP*^, *tau*^*+/GFP*^ and *tau*^*+/+*^ mice or both genders at 16 months of age.

To determine the long-term effect of tau reduction and/or depletion *in vivo*, we crossed mice heterozygous green fluorescence protein (GFP) knockin into exon 1 of the murine *Mapt* gene [7] (Fig 1A) with each other to obtain heterozygous (*tau*^*GFP/+*^) and homozygous (*tau*^*GFP/GFP*^) offspring and wild-type (*tau*^*+/+*^) littermates as controls at Mendelian distribution (Fig 1B and 1C), and aged the mice to an advanced age of 19–22 months. Inclusion of a stop codon after the GFP open reading frame resulted in expression of a fusion protein containing the first 32 amino acids of tau followed by the GFP sequence, and hence, depletion of all functional domains of tau. As a consequence tau levels were reduced in heterozygous *tau*^*GFP/+*^ and absent in homozygous *tau*^*GFP/GFP*^ mice, as determined by immuoblotting of hippocampal tissue extracts (Fig 1D). Neither *tau*^*GFP/+*^ nor *tau*^*GFP/GFP*^ mice of both genders showed differences in body weight compared to *tau*^*+/+*^ littermates at 16 months of age, suggesting no gross morphological differences (Fig 1E).

**Fig 2:**
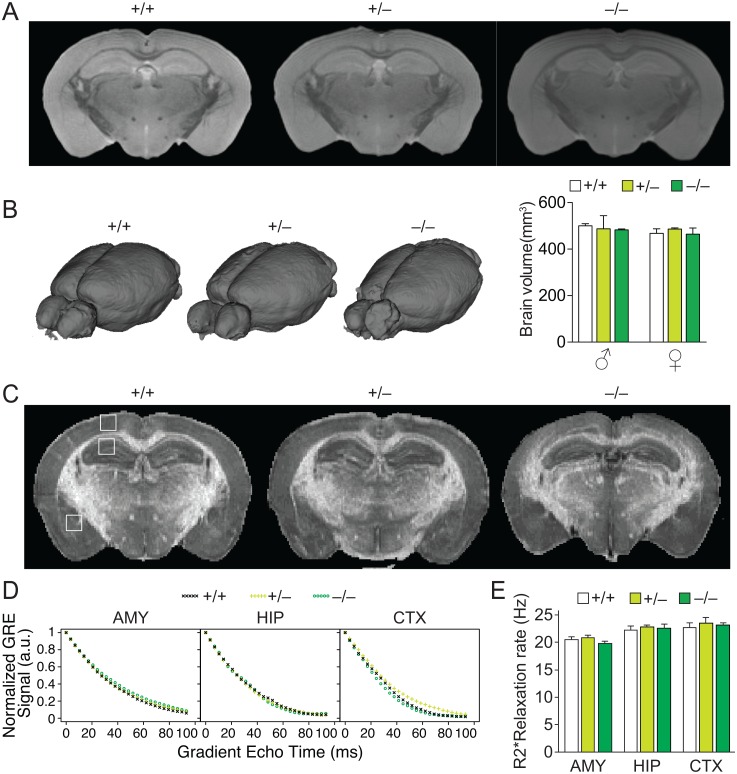
Normal brain imaging in aged *tau*^*GFP/GFP*^ mice. (A) Representative coronal MRI scan showing no morphological changes in 19–20 month-old *tau*^*GFP/GFP*^ (–/–) and *tau*^*+/GFP*^ (+/–) compared to *tau*^*+/+*^ (+/+) mice. (B) Whole organ rendering of MRI brain scans from *tau*^*GFP/GFP*^, *tau*^*+/GFP*^ and *tau*^*+/+*^ mice, revealed unchanged brain volumes in *tau*^*GFP/GFP*^ and *tau*^*+/GFP*^ compared to *tau*^*+/+*^ mice (n = 3/genotype/gender). (C) Representative R2* relaxation images of *tau*^*GFP/GFP*^, *tau*^*+/GFP*^ and *tau*^*+/+*^ MRI scans. White boxes demark area used for determining R2* relaxation extinction co-efficient in amygdala, hippocampus and cortex (see D). (D) Comparable R2* relaxation curves for amygdala (AMY), hippocampus (HIP) and cortex (CTX) in *tau*^*GFP/GFP*^, *tau*^*+/GFP*^ and *tau*^*+/+*^ mice. (E) R2* relaxation rates were indistinguishable in AMY, HIP and CTX of *tau*^*GFP/GFP*^, *tau*^*+/GFP*^ and *tau*^*+/+*^ mice (n = 6).

To assess any overt morphological changes of the brain, we analyzed *tau*^*GFP/GFP*^, *tau*^*GFP/+*^ and *tau*^*+/+*^ littermate mice of both genders by magnetic resonance imaging (MRI) (Fig 2A). This did not reveal any morphological differences between the genotypes and, accordingly, total brain volumes were indistinguishable between the groups (Fig 2B). Since Lei and colleagues reported a significant accumulation of iron in the brains of their *tau*^*–/–*^strain after 12 months of age [11], we used R2* relaxation mapping (Fig 2C), as an MRI biomarker of iron accumulation. However, R2* relaxation curves and their extinction co-efficient in amygdala, hippocampus and cortex were similar in all genotype from our *tau*^*GFP/GFP*^ strain at 19–20 months of age, suggesting no abundance of iron in their brains (Fig 2D and 2E).

### *Tau*^*GFP/GFP*^ mice have no functional deficits

Next, to determine if long-term reduction and/or depletion of tau resulted in functional deficit, we subjected 16 months-old *tau*^*GFP/GFP*^, *tau*^*GFP/+*^ and *tau*^*+/+*^ mice to a battery of motor, memory and behavioral tests. Groups of female and male mice were analyzed together and performance of the different genotypes also compared.

**Fig 3:**
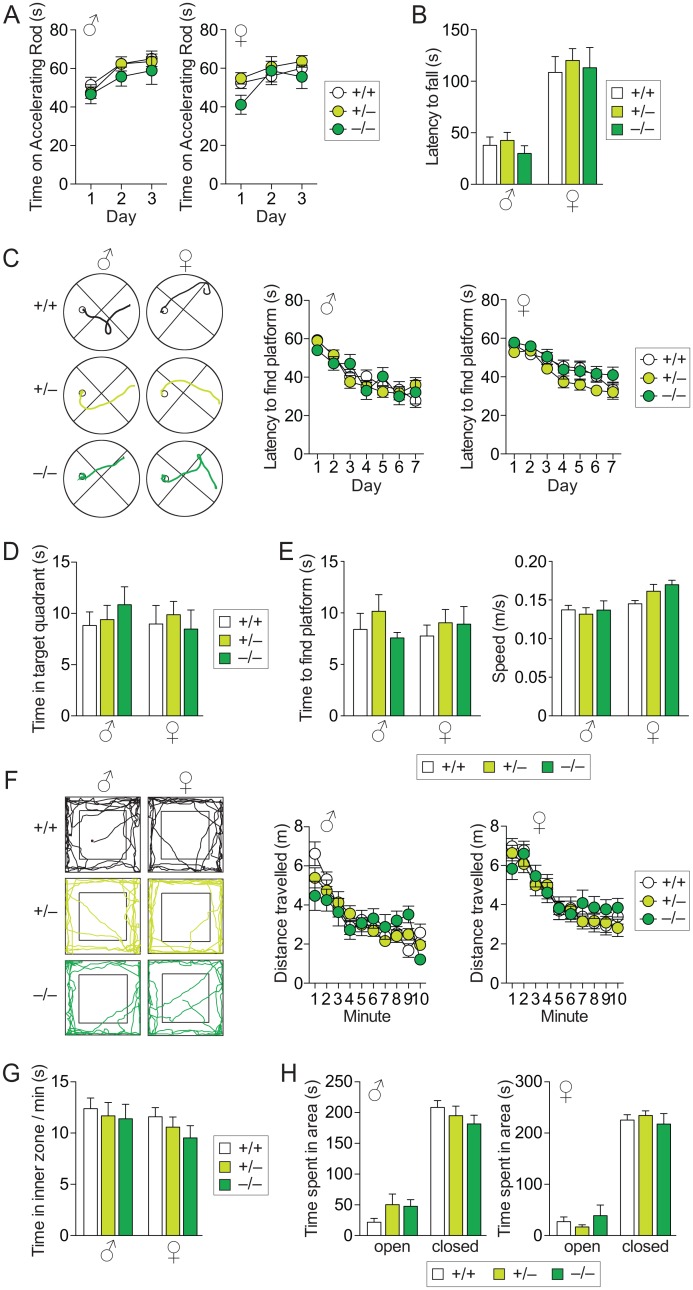
Absence of motor, memory and behavioral deficits in aged *tau*^*GFP/GFP*^ mice. Eight male and 12 female *tau*^*GFP/GFP*^ (–/–), 18 male and 20 female *tau*^*GFP/+*^ (+/–), and 12 male and 15 female *tau*^*+/+*^ (+/+) mice were tested, unless otherwise stated; (A) Similar latencies to fall on the accelerating Rota-Rod presented by *tau*^*GFP/GFP*^, *tau*^*+/GFP*^ and *tau*^*+/+*^ mice of both genders over 3 test days. (B) Comparable latency to fall off the inverted wire suggesting similar grip strength in *tau*^*GFP/GFP*^, *tau*^*+/GFP*^ and *tau*^*+/+*^ mice. (C) Examples of swim paths in the Morris water maze from *tau*^*GFP/GFP*^, *tau*^*+/GFP*^ and *tau*^*+/+*^ mice (small circle indicates position of submerged escape platform). Similar improvements in the latency to find the submerged platform in female and male *tau*^*GFP/GFP*^, *tau*^*+/GFP*^ and *tau*^*+/+*^ mice suggesting normal memory formation (1 female *tau*^*GFP/GFP*^, 2 female *tau*^*+/GFP*^ and 4 female *tau*^*+/+*^ mice were excluded due to floating behavior). (D) Both, female and male *tau*^*GFP/GFP*^, *tau*^*+/GFP*^ and *tau*^*+/+*^ mice spent comparable times in the target quadrant during probe trials. (E) All mice found the marked platform similarly fast and average swim speed during MWM testing was comparable between genotypes, suggesting normal vision and swimming performance. (F) Examples of exploration paths of *tau*^*GFP/GFP*^, *tau*^*+/GFP*^ and *tau*^*+/+*^ mice in the open field arena during the first minute of 10 minute trials. Activity of all mice were comparable, as determined by distance travelled per minute. (G) No differences in time spent in the inner zone during OF testing of *tau*^*GFP/GFP*^, *tau*^*+/GFP*^ and *tau*^*+/+*^ mice. (H) During elevated plus maze testing, both female and male *tau*^*GFP/GFP*^, *tau*^*+/GFP*^ and *tau*^*+/+*^ mice predominantly spent time in the closed arms, with no differences between genotypes, suggesting no changes in anxiety/disinhibition.

First, we assessed motor performance, by testing 16 month-old *tau*^*GFP/GFP*^ and their *tau*^*GFP/+*^ and *tau*^*+/+*^ littermates on the accelerating-mode Rota-Rod. Over three consecutive days, both female and male mice of all genotypes presented the same performance, suggesting that motor co-ordination is unaffected in aged mice of our *tau*^*GFP/GFP*^ strain (Fig 3A). Similarly, the latency to fall off the inverted grid during hanging wire testing was indistinguishable between *tau*^*GFP/GFP*^ and their *tau*^*GFP/+*^ and *tau*^*+/+*^ littermates of both genders, suggesting comparable body strength and co-ordination (Fig 3B). We also attempted to test the mice with the vertical pole test, but mice of all genotypes and genders failed to hold onto the pole and slid to the bottom as soon as they were released. We previously reported this test with younger mice of different genotypes, including motor impaired strains [16, 17], suggesting that *tau*^*GFP/GFP*^ and their *tau*^*GFP/+*^ and *tau*^*+/+*^ littermates at an age of 16 month were incapable, rather than problems with the test setup or equipment.

Next, we determined if memory formation was compromised in aged *tau*^*GFP/GFP*^ or *tau*^*GFP/+*^ mice. Therefore, we assessed their spatial memory abilities using the standard Morris Water Maze paradigm. A similar progressive reduction in the latency to escape onto the submerged platform over seven consecutive testing days, suggested normal memory formation in both male and female *tau*^*GFP/GFP*^, *tau*^*GFP/+*^ and *tau*^*+/+*^ mice (Fig 3C). During probe trials, again, there was no significant difference in the time spent in the target platform quadrant between mice of all genotypes for both genders (Fig 3D). All mice found the platform rapidly when it was equipped with a flag at comparable speeds during MWM testing, suggesting normal vision and ability to swim (Fig 3E).

Finally, we tested locomotor and anxiety/disinhibition behavior using the Open Field (OF) and the Elevated Plus Maze (EPM) tasks [16]. When exposed to a novel environment in the open field arena, *tau*^*GFP/GFP*^ mice showed no difference in activity or time spent in the inner zone compared to their *tau*^*GFP/+*^ and *tau*^*+/+*^ littermates, suggesting normal exploration behavior and absence of hyperactivity (Fig 3F and 3G). Likewise, *tau*^*GFP/GFP*^ mice showed no difference in anxiety/disinhibition behavior compared to their *tau*^*GFP/+*^ and *tau*^*+/+*^ littermates, and spent comparable times in the open arms of the EPM (Fig 3H). Taken together, these results show that both long-term reduction and deletion of tau *in vivo* does not compromise motor co-ordination, muscle strength, spatial memory or anxiety/disinhibition behavior in *tau*^*GFP/+*^ or *tau*^*GFP/GFP*^ mice, compared to their *tau*^*+/+*^ littermates.

### *Tau*^*GFP/GFP*^ mice show no substantia nigra cell loss

**Fig 4:**
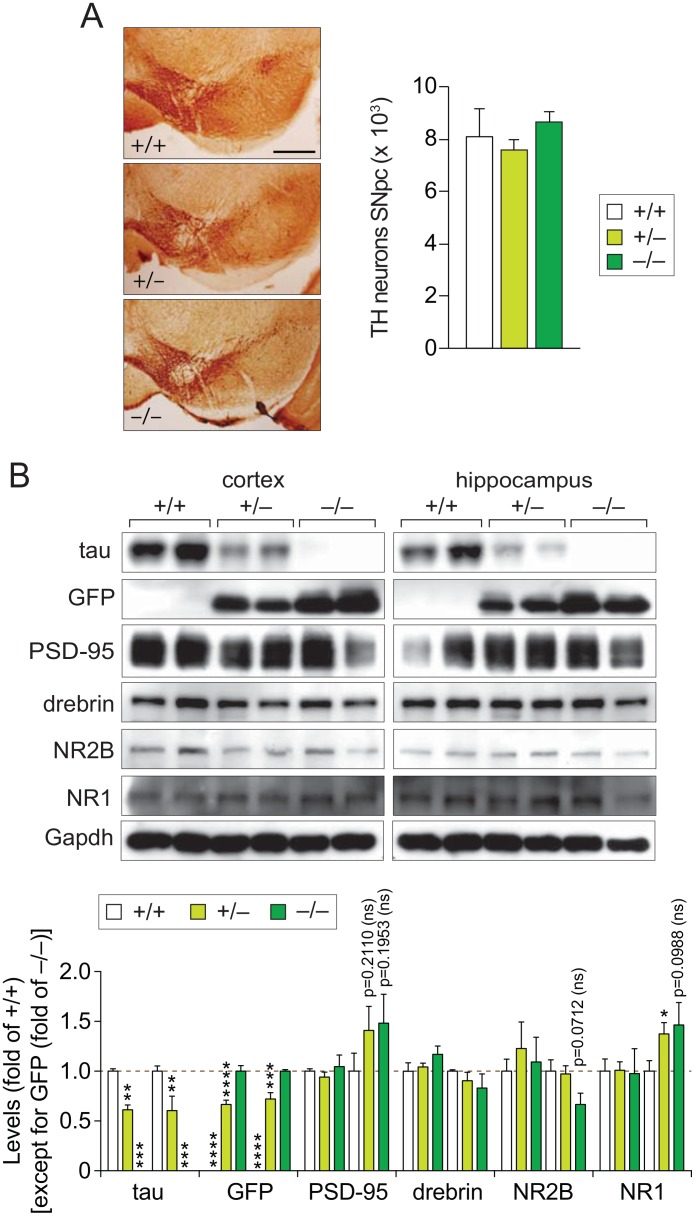
No loss of substantia nigra neurons and synaptic proteins in aged *tau*^*GFP/GFP*^ mice. (A) Stereological assessment of serial coronal sections of *tau*^*GFP/GFP*^ (–/–), *tau*^*+/GFP*^ (+/–) and *tau*^*+/+*^ (+/+) brains stained with a tyrosine hydroxylase (TH)-specific antibody revealed indistinguishable numbers of dopaminergic SN neurons (n = 5/genotype). Representative stainings are shown. (B) Western blotting of brain extracts from cortex and hippocampus of 20 month-old *tau*^*GFP/GFP*^, *tau*^*+/GFP*^ and *tau*^*+/+*^ mice showed no significant changes in levels of PSD-95, drebrin, NR1 and NR2B, and due to reduction of tau levels (and expression of GFP). Gapdh was probed to confirm equal loading. Quantification of blots was done from independent animals (n = 5; One-way ANOVA: **P<0.01, ***P<0.001, ****P<0.0001; Student’s t-test: *P<0.05 vs WT; ns, not significant (*P*-values provided)).

Motor deficits in another *tau*^*–/–*^strain have been associated with a reduction in substantia nigra pars compacta (SNpc) neurons [11, 14]. Although *tau*^*GFP/GFP*^ mice did not present with comparable motor deficits (Fig 3), we determined if the number of tyrosine hydroxylase (TH) positive SNpc neurons were altered upon long-term reduction and/or depletion of tau in our mice. However, stereological analysis of serial sections stained with antibodies to TH did not reveal differences in numbers of SNpc neurons between *tau*^*GFP/GFP*^, *tau*^*GFP/+*^ and *tau*^*+/+*^ mice (Fig 4A). Hence, tau reduction had no effects on SNpc neurons numbers in *tau*^*GFP/GFP*^ or *tau*^*GFP/+*^ mice.

### *Tau*^*GFP/GFP*^ mice present with unaltered levels of synaptic proteins

To determine if levels of synaptic proteins are affected by long-term reduction and/or depletion of tau *in vivo*, we next analyzed protein extracts from brains of 22 months-old *tau*^*GFP/GFP*^, *tau*^*GFP/+*^ and *tau*^*+/+*^ mice, focusing on post-synaptic proteins and glutamatergic receptor components. Levels of NR subunits NR1 and NR2B, as well as of PSD-95 and the post-synaptic protein drebrin were indistinguishable in the hippocampus of aged *tau*^*GFP/GFP*^, *tau*^*GFP/+*^ and *tau*^*+/+*^ mice of both genders (Fig 4B). This suggests that long-term depletion of tau does not compromise NR levels in *tau*^*GFP/GFP*^ mice, in line with absence of functional and morphological deficits at advanced ages in this strain.

## Discussion

In the present study, we have found no functional deficits in both female and male *tau*^*GFP/GFP*^ or *tau*^*GFP/+*^ mice at 16 months of age, when compared to their *tau*^*+/+*^ littermates; this included motor (Rota-Rod, Hanging Wire), spatial memory (MWM), locomotor (OF) and anxiety/disinhibition behavior (EPM) testing. Furthermore, *tau*^*GFP/GFP*^, *tau*^*GFP/+*^ and *tau*^*+/+*^ brain were indistinguishable using MRI analysis at 22 months of age. At the cellular and molecular level, we found no changes to TH-positive SNpc neuron numbers, synaptic morphology and density and expression levels of post-synaptic protein, including NR subunits.

Four different *tau*^*–/–*^lines have been reported in the literature to date [2]. The first one was introduced in 1994 by Harada and colleagues (referred to as *H*.*tau*^*–/–*^throughout the discussion to differentiate the individual lines), reporting no overt phenotypes, but some alterations in tubulin spacing at the microtubule level and indicating increased Map1B levels to compensate for the absence of tau [5]. The latter was substantiated by crossing *tau*^*–/–*^with *Map1b*^*–/–*^mice, resulting in a detrimental developmental phenotype with inhibition of axonal elongation and reduced neuronal migration [9]. In 2001, Dawson and colleagues reported a new *tau*^*–/–*^line (*D*.*tau*^*–/–*^) with mild axonal outgrowth deficits in culture neurons, but no overt phenotype [6]. At the same time, Tucker and colleagues developed a *tau*^*–/–*^line (*tau*^*GFP/GFP*^) with knockin of GFP into exon 1 of *Mapt*, resulting in depletion of the tau protein [7]. This line was developed to isolate phenotypically normal heterozygous *tau*^*GFP/+*^ neurons from brains by fluorescence-activated cell sorting for cultures. Finally, in 2007, Fujio and colleagues reported a *tau*^*–/–*^line (*F*.*tau*^*–/–*^) that awaits further characterization [8].

The initial report of absence of overt deficits in all *tau*^*–/–*^lines was followed by a number of reports in more recent years that reported both absence and presence of functional deficits and morphological/molecular changes even when using the same *tau*^*–/–*^lines, creating an ongoing controversy in the field. Following the first report of the *H*.*tau*^*–/–*^line [5], muscle weakness and deficits in motor coordination together with hyperactivity in the open field task and contextual fear conditioning learning impairments in 3 month-old mice of this line were reported [10]. This was followed by a series of studies that reported normal spatial memory in the MWM, radial arm maze and Y-maze, using two different *tau*^*–/–*^strains at young (3–7 months; *D*.*tau*^*–/–*^and *tau*^*GFP/GFP*^) [3, 13], medium (12 months; *D*.*tau*^*–/–*^) [22] and, more recently, advanced ages (22 months; *D*.*tau*^*–/–*^) [12]. However, there are also reports of memory deficits in these *tau*^*–/–*^lines, including spatial memory deficits found in *tau*^*GFP/GFP*^ mice at 15 months of age [14], delays in memory acquisition in the MWM (without deficits in subsequent probe trials) and reduced contextual fear conditioning learning in 6 month-old male *tau*^*GFP/GFP*^ mice [23]. The latter study is in contrast to our findings of normal MWM performance in *tau*^*GFP/GFP*^ mice of both genders at advanced ages of over 16 months. In contrast, a recent study reported memory deficits in the Y-maze in *D*.*tau*^*–/–*^mice at 12 months of age on a mixed C57B6/129Sv, but not a pure C57Bl/6 background [24]. Interestingly, young *D*.*tau*^*–/–*^mice at 4 months of age were found to have changes in wave patterns during EEG recordings, possibly suggesting altered interneuron function [25]. However, we found no changes in field recordings from *tau*^*GFP/GFP*^ mice at 3 months of age [3]. Similar to the findings on memory deficits of *tau*^*–/–*^mice, reports on motor deficits presented variable findings; accordingly, findings of virtually unaffected motor performance during Rota-Rod and/or pole testing of *D*.*tau*^*–/–*^mice at 5 [26], 12 [22] and 22–24 months of age [12, 27], were contrasted by reports of impaired Rota-Rod and pole test performance of 12 month-old *D*.*tau*^*–/–*^mice [11]. The latter study received some support by another group showing mild motor deficits in *tau*^*GFP/GFP*^ at 9 months of age that did not, however, progress as mice became older [14]. In addition, muscle weakness, tested on the hanging wire, and impaired motor coordination, assessed by beam crossing, were reported for *H*.*tau*^*–/–*^mice [10], and to a lesser degree in *D*.*tau*^*–/–*^mice [12, 26]. Yet, a recent study reported motor deficits on the Rota-Rod, hanging wire and reduced grip strength together with sciatic nerve pathology in old (17–22 months), but not young (4–6 months) *D*.*tau*^*–/–*^mice [28]. In the present study, we found no motor deficits or muscle weakness in *tau*^*GFP/GFP*^ mice during Rota-Rod or hanging wire testing. Increased locomotor activity was reported in *H*.*tau*^*–/–*^mice [10], while in *D*.*tau*^*–/–*^mice open field recordings showed unchanged (22 months) [12] or increased (12 months) [24] levels of activity. Activity of *tau*^*GFP/GFP*^ mice was found to be normal at 6 months of age [23], which is in line with the findings of wild-type-like activity of aged *tau*^*GFP/GFP*^ mice in the open field test in the present study. At the morphological and molecular level, brain atrophy with enlargement of ventricles, iron accumulation and loss of TH-positive SNpc neurons were found in 12 months-old *D*.*tau*^*–/–*^mice [11]. The loss of TH neurons was confirmed in 17 month-old *tau*^*GFP/GFP*^ mice by one group [14], while no changes in TH were found in the *D*.*tau*^*–/–*^line by another group [12]. Finally, one report revealed a significant reduction of NR subunits and post-synaptic proteins in aged *tau*^*GFP/GFP*^ mice [14]. To this end, we found no changes in the numbers of TH-positive neurons in the SNpc, synaptic density or of levels of post-synaptic proteins, including NR subunits in aged *tau*^*GFP/GFP*^ mice. Furthermore, MRI analysis showed absence of gross morphological changes in *tau*^*GFP/GFP*^ mice, and R2* relaxation analysis indicated no overt iron accumulation in their brains.

In summary, the effects of short- and long-term depletion of tau found by different groups vary, even if the same *tau*^*–/–*^strains are used. This variability suggests a number of possible confounding factors, such as differences in the gene targeting approach and/or genetic backgrounds used, the use of separately bred controls instead of littermates (leading to potential differences due to genetic backgrounds rather than the actual *tau*^*–/–*^), differences in behavioral testing procedures, the variability of standard chows providing different nutrients and likely differences in housing conditions/regulations (such as the use of isolated cage systems, numbers of littermates per cage and levels of enrichment). It appears hard to pinpoint the ‘one’ factor causing these alterations in the phenotypes of *tau*^*–/–*^mice, although some studies provided first clues. For example, the supplementation of the chow with 6.0g/kg omega-3 fatty acid prevented the deficits in one cohort of *D*.*tau*^*–/–*^mice [14], pointing at differences in chow formula as a confounding factor. Interestingly, *D*.*tau*^*–/–*^mice reported to have no deficits were maintained on a diet containing 3.3g/kg omega-3 facts acid [12], while another colony of *D*.*tau*^*–/–*^mice with motor deficits and SNpc neuron loss were kept on a chow without or with only 0.7g/kg omega-3 fatty acid [24]. The standard diet used in this study contained 3.5g/kg omega-3 fatty acid derived from fishmeal, which may contribute to the absence of deficits in our *tau*^*GFP/GFP*^ colony. However, our *tau*^*GFP/GFP*^ colony was previously maintained on a chow containing only 0.5g/kg omega-3 fatty acid, without overt motor phenotype and memory deficits [3]. On the other hand, differences in the genetic background did not alter the impairments in a *D*.*tau*^*–/–*^cohort [24], which may suggest a less prominent role of the background used in this context. Nevertheless, the differences in findings between sites, strains and cohorts highlights the importance of thorough characterization of the phenotypes of *tau*^*–/–*^mice at each individual laboratory to avoid erroneous conclusions, until the confounding factor(s) causing this variability have been identified and validated.
